# Association of Maternal Erythrocyte PUFA during Pregnancy with Offspring Allergy in the Chinese Population

**DOI:** 10.3390/nu14112312

**Published:** 2022-05-31

**Authors:** Shanshan Peng, Zhicheng Du, Yannan He, Feng Zhao, Yujing Chen, Shengchi Wu, Yuantao Hao, Li Cai

**Affiliations:** 1 Department of Medical Statistics, School of Public Health, Sun Yat-sen University, Guangzhou 510080, China; pengshsh5@mail2.sysu.edu.cn (S.P.); duzhch5@mail.sysu.edu.cn (Z.D.); 2 Institute of Nutrition & Health, Qingdao University, Qingdao 266071, China; aprilho328@126.com (Y.H.); fengzhao21c@163.com (F.Z.); 3 OmegaBandz. Inc., Shanghai 201815, China; 4 Department of Maternal and Child Health, School of Public Health, Sun Yat-sen University, Guangzhou 510080, China; chenyj336@mail2.sysu.edu.cn (Y.C.); wushch3@mail2.sysu.edu.cn (S.W.); 5 Peking University Center for Public Health and Epidemic Preparedness & Response, Beijing 100191, China; 6 Guangdong Provincial Key Laboratory of Food, Nutrition and Health, School of Public Health, Sun Yat-sen University, Guangzhou 510080, China

**Keywords:** allergic disease, polyunsaturated fatty acids, offspring, pregnancy

## Abstract

Findings on prenatal polyunsaturated fatty acids (PUFA) and offspring allergies have been inconsistent, and the majority of studies have focused on Western populations. This study aimed to investigate the associations between maternal erythrocyte PUFA and offspring allergies in the first 2 years in the Chinese population. We included 573 mother–infant pairs from a birth cohort. Based on the outpatient medical records, we identified the diagnosis and time of offspring allergic disease onset. We measured erythrocyte fatty acids by gas chromatography. Associations were examined using Cox regression. We found that higher maternal total PUFA levels (*HR* = 0.80; 95% *CI*: 0.68, 0.94), especially of arachidonic acid (AA) (*HR* = 0.79; 95% *CI*: 0.65, 0.97) and *n*-3 PUFA (*HR* = 0.77; 95% *CI*: 0.62, 0.97), were associated with reduced risk of offspring allergies. Similar results were found for eczema. Compared with children without a maternal allergy history, the associations of total PUFA (*p* = 0.028) and *n*-6 PUFA (*p* = 0.013) with offspring allergies were stronger in those with a maternal allergy history. Maternal erythrocyte total PUFA, especially AA, and *n*-3 PUFA were inversely associated with offspring allergies within 2 years of age. There was a significant interaction between maternal allergy history and maternal PUFA in offspring allergies.

## 1. Introduction

The prevalence of allergic diseases has risen dramatically worldwide in recent decades, aggravating the economic burden on families and society [1,2,3]. Consequently, early prevention of allergic diseases has been increasingly emphasized.

The Developmental Origin of Health and Disease (DOHaD) theory suggests that early life is a critical window of development that will influence the occurrence of future diseases [4]. Previous studies have shown that the embryo is in a critical period of susceptibility when the intrauterine nutrition can affect the development and maturity of the immune system, thereby irreversibly affecting the development of allergies [5,6,7]. 

In 1997, Black et al. first linked the increased prevalence of allergic diseases in recent years to the changes in dietary fat intake, notably an increase in *n*-6 polyunsaturated fatty acids (*n*-6 PUFA), and a decrease in *n*-3 PUFA [8,9]. Of note, the PUFA can be easily transferred across the placenta [10]. Studies have linked maternal prenatal PUFA to the development of allergic diseases. In general, *n*-6 PUFA is associated with pro-inflammatory responses, while *n*-3 PUFA shows an opposite association [11,12]. Contrarily, the Dutch Generation R study showed that maternal *n*-6 PUFA but not *n*-3 PUFA was associated with a reduced risk of childhood asthma [13], whereas other studies found a weak or no association between prenatal PUFA and offspring allergies [14,15]. The evidence regarding the effect of PUFA in early life on the risk of allergic diseases has been controversial. It is worth noting that the biosynthetic capacity of PUFA is mainly determined by the rate-limiting enzymes, which are encoded by genes closely associated with ethnicity [16]. On top of this, the effect of *n*-3 PUFA on allergic diseases may vary by ethnicity because of genetic differences [17,18]. 

There are two gaps in the existing literature. First, the majority of studies reported in the literature focused on Western populations [13,14], thus highlighting the need for more study in Asian populations. Second, previous studies have mostly used plasma fatty acids or food intake of PUFA as biomarkers, rather than erythrocyte fatty acids. Erythrocyte fatty acids are considered more accurate and objective than FFQ [19]. Compared to plasma fatty acids, erythrocyte fatty acids are not affected by fasting [20,21]. Therefore, this study aimed to assess the prospective association of maternal erythrocyte PUFA with offspring allergies in the Chinese population.

## 2. Materials and Methods

### 2.1. Study Design and Population

The present study subjects were mother–infant pairs from an ongoing prospective longitudinal birth cohort (ClinicalTrial.gov number: NCT03023293). For this study, we enrolled pregnant women (20–28 weeks’ gestation) aged 20–45 years at Yuexiu district maternal and child health hospital in Guangzhou, China. Individuals with pre-existing diabetes mellitus, cardiovascular disease, thyroid disease, hematopathy, polycystic ovary syndrome, pregnancy-related infection, mental disorder or multiple pregnancies were excluded from the study. Additionally, those pregnant women who did not have blood samples at baseline of the survey were also excluded.

In total, 592 mother–newborn pairs were recruited between March 2017 to November 2018. We further excluded those without allergy information in offspring at age 2 (*n* = 19). Therefore, 573 mother–child pairs were included in the final analysis. The Ethics Committee of the School of Public Health, Sun Yat-Sen University approved this study. All participants were carefully instructed and signed an informed consent at initial enrollment.

### 2.2. Laboratory Analysis

Whole blood, collected from women at 24–28 weeks of gestation who had fasted at least 10 h, was centrifuged. The red cell concentrate was kept at −80 °C until later laboratory analysis. The quantitative measurement of erythrocyte fatty acids was carried out by gas chromatography. 

The red cell samples were removed from the refrigerator and thawed. Tris-HCl buffer was added to the sample. After the red blood cells were hemolyzed, we centrifuged them at 3000 rpm/10 min to obtain the bottom layer of milky red blood cell fragments. The lipid component of erythrocyte fragments was extracted with a chloroform–methanol (2:1, *v*/*v*) solvent system containing 10 mg/L of butylated-hydroxytoluene (BHT, Sigma Chemical Co., St. Louis, MO, USA) [22]. Fatty acid methyl esters from lipid extract were transesterified with H_2_SO_4_ in methanol (5%, *v*/*v*), together with toluene, for 2 h at 70 °C in sealed tubes. The methanol layer was transferred to a new test tube, blown by nitrogen, and then dissolved in hexane. The derived fatty acid methyl esters (FAME) were analyzed with a Shimadzu GC-14C (Shimadzu Corporation, Kyoto, Japan) fitted with a flame ionization detector (FID) and a 60 m * 0.25 mm (i.d.) * 0.25 μm (film thickness) fused silica bonded phase column (DB-23, Agilent Corporation, Santa Clara, CA, USA). Nitrogen was the carrier gas at 300 kPa pressure. The temperatures of the injector and detector were both 270 °C. The column temperature was programmed from 150 to 180 °C at a rate of 10 °C/min, with an initial holding time of 2 min; then, it was further increased to 215 °C at a rate of 2.5 °C/min and held for 6 min. Finally, the temperature was increased to 230 °C at 10 °C/min and kept for another 5 min.

Identification of fatty acids was carried out by comparing retention time with standard mixtures of fatty acid methyl ester (Nu-Chek Prep, Inc., Waterville, MN, USA). The quantification of fatty acid compositions was achieved by comparing the peak areas with internal standard (Tricosanoic acid, C23:0), which was added to the samples (500 mg sample contained 1 mg internal standard) prior to extraction [22]. 

### 2.3. Fatty Acids Calculations

Specific fatty acids were expressed as fractions (%) of their peak area compared to the total peak area of all fatty acids. Consequently, *n*-6 PUFA, arachidonic acid (AA), linoleic acid (LA), *γ*-linolenic acid (GLA), *dihomo*-*γ*-linolenic (DGLA), *n*-3 PUFA, *α*-linolenic acid (ALA), eicosapentaenoic acid (EPA), docosahexaenoic acid (DHA) and docosapentaenoic acid (DPA) were reported as a percentage of total fatty acids. Omega-3 index was calculated as ((erythrocyte EPA + erythrocyte DHA)/total erythrocyte fatty acids) × 100% [23]. The *n*-6: *n*-3 ratio was determined by dividing total *n*-6 PUFA (%) by total *n*-3 PUFA (%). Similarly, the AA: EPA ratio was calculated by dividing AA (%) by EPA (%). 

### 2.4. Ascertainment of Offspring Allergic Disease

Based on outpatient medical records provided by parents, we identified the onset time and diagnosis of allergic diseases in offspring within 2 years of age, including eczema, food allergy, urticaria, allergic dermatitis, allergic rhinitis, allergic conjunctivitis, pollen allergy and asthma. During interviews with parents, we also collected detailed information on allergies to validate diagnosis via standardized questions adapted from the International Study of Asthma and Allergies in Childhood (ISAAC) questionnaire [24]. In our study, any allergic disease was defined as any one or more of eczema, food allergy, urticaria, allergic dermatitis, allergic rhinitis, allergic conjunctivitis, pollen allergy or asthma up to the age of 2 years.

### 2.5. Assessment of Covariates

At baseline survey, the demographics and lifestyle factors during pregnancy were investigated through face-to-face interviews, including maternal age, educational level, occupation, monthly household income, frequency of passive smoking and alcohol consumption. Height (nearest 0.1 cm) was measured by trained clinical nurses, and pre-pregnancy weight was self-reported. Pre-pregnancy body mass index (BMI, kg/m^2^) was calculated as pre-pregnancy weight (kg) divided by height squared (m^2^).

Information on children’s gender, breastfeeding duration, the introduction of solid food in 6 months and maternal allergy history were collected at the ages of 6 months and 2 years by structured questionnaire.

### 2.6. Statistical Analysis

Demographic characteristics and maternal erythrocyte fatty acids of the study population were described as proportions for categorical variables or median (Q1, Q3) for continuous variables. Differences among groups were tested using chi-square test or Wilcoxon rank-sum test. 

Cox regression was utilized to evaluate the associations between maternal erythrocyte fatty acids and offspring allergies by calculating the hazard ratios (*HRs*) and 95% confidence interval (95% *CI*). The main model was adjusted for maternal age, pre-pregnancy BMI, monthly household income, educational level, occupation, child’s gender, breastfeeding duration, maternal allergy history, complementary feeding time, maternal passive smoking and alcohol consumption during pregnancy.

We further conducted sensitive analyses and stratified analyses. In the sensitivity analysis, we studied the associations between maternal erythrocyte fatty acids and specific offspring allergies, including eczema, food allergy, urticaria, and allergic rhinitis. After that, we compared differences between subgroups by stratifying maternal allergy history and maternal age, respectively. Meanwhile, the model was adjusted for potential confounding factors consistent with the main model (stratified factors excluded). All analyses were carried out by using R Version 4.0.5 with statistical significance set at 0.05.

## 3. Results

### 3.1. Characteristics of Study Participants

**Figure 1:**
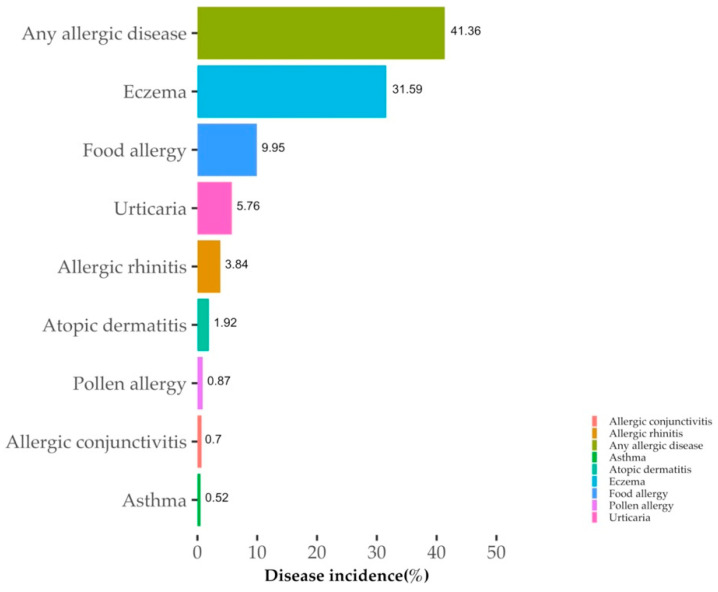
The incidence of specific allergic disease in offspring within the first 2 years.

The general characteristics of the participants are presented in Table 1. During the first 2 years after birth, 237 out of 573 children (41.36%) had an allergic disease. The incidence of eczema was as high as 31.59%, followed by the incidence of food allergy at 9.95% (Figure 1). Compared to children without an allergy, those with an allergy tended to have a shorter breastfeeding duration (*p* = 0.034). Their mothers were also more likely to have an allergy history (*p* = 0.004). Among mothers of kids with allergies, a higher proportion were exposed to passive smoking during pregnancy than mothers of those without allergies (*p* = 0.037). No significant differences were observed in other characteristics between the allergy and non-allergy groups.

### 3.2. Maternal Erythrocyte Fatty Acids

Table 2 describes the distribution of maternal erythrocyte fatty acids during pregnancy in the allergy and non-allergy groups. Compared with mothers of children without allergies, mothers of children with allergies had lower levels of erythrocyte total PUFA (*p* = 0.044), AA (*p* = 0.042), and *n*-3 PUFA (*p* = 0.011) during pregnancy. There was no significant difference between the allergy and non-allergy groups among other fatty acids.

### 3.3. Associations of Maternal Erythrocyte Fatty Acids with Offspring Allergic Disease

As shown in Table 3, the associations between maternal erythrocyte fatty acids during pregnancy and offspring allergies was analyzed by Cox regression. After adjustment for potential confounders, maternal erythrocyte total PUFA (*HR* = 0.80; 95% *CI*: 0.68, 0.94), AA (*HR* = 0.79; 95% *CI*: 0.65, 0.97), and *n*-3 PUFA (*HR* = 0.77; 95% *CI*: 0.62, 0.97) were adversely associated with offspring allergies. Similar results were found for eczema in offspring (Supplementary Table S1). Non-significant associations were observed between other fatty acids and any allergic disease. 

We also analyzed the associations between maternal fatty acids and different types of allergic diseases in offspring. The results also indicated a negative association between maternal AA (*HR* = 0.65; 95% *CI*: 0.44, 0.96) and food allergy in children. There were no statistically significant associations between maternal fatty acids and urticaria or allergic rhinitis in offspring (Supplementary Table S1).

### 3.4. Stratified Analysis

As shown in Table 4, we carried out a stratified analysis of maternal allergy history and maternal age for the association between maternal erythrocyte fatty acids and offspring allergies. Compared with children without maternal allergy history, we found that the associations of total PUFA (*p* = 0.028), *n*-6 PUFA (*p* = 0.013), LA (*p* = 0.006) and DPA (*p* = 0.015) with allergies were stronger in those with a maternal allergy history. However, no significant interlayer difference was found in the associations of other fatty acids and offspring allergies. In addition, we also found a non-significant difference between groups in the maternal age stratified analysis.

## 4. Discussion

To the best of our knowledge, this is the first study reporting an association of maternal erythrocyte PUFA with offspring allergic disease in an Asian population. We found that higher concentrations of maternal erythrocyte total PUFA, and especially of AA and *n*-3 PUFA, were associated with a decreased risk of offspring allergy at 2 years of age. In sensitivity analyses for specific allergic diseases, we obtained similar findings only for eczema in children, which may be attributed to the highest incidence of eczema in our study population. Allergies in infants and young children usually go through a complex natural process [25], often initiated by eczema and food allergy, with the highest incidence before the age of 3 years. In contrast, most allergic diseases, such as asthma and allergic rhinitis, tend to appear in children aged 5 years and older. Overall, our results were consistent with the atopic march. Interestingly, the inverse associations of maternal erythrocyte total PUFA, *n*-6 PUFA, and LA with offspring allergic diseases were more significant in those with a maternal allergy history.

Our results indicated that mothers with higher concentrations of erythrocyte total PUFA, especially *n*-3 PUFA, have a lower risk of offspring allergy. These findings were consistent with the results of three prospective cohort studies conducted in Sweden and the USA [26,27,28]. These studies found significant associations between maternal *n*-3 PUFA and lower risk of allergic diseases in children, and we extended similar findings to the Chinese population. Mechanistically, PUFA as an important component of membrane phospholipids, can modulate immunologic function by affecting eicosanoids production, cell membrane fluidity and gene expression [29,30,31]. Particularly, *n*-3 PUFA can enhance or inhibit multiple stages in classic Th1/Th2 allergic reactions through the effects on cell membrane permeability, cellular signal transduction and gene transcription, such as inhibiting the transcription factor NF-κB, thereby inhibiting the development of allergic diseases [32,33]. Although many studies have shown that higher *n*-3 PUFA during pregnancy can reduce the risk of allergies in children, some studies have shown mixed results [34,35]. Contradictory findings of prior studies may partly be explained by the heterogeneity in tools used to assess PUFA level, outcome definitions, the timing of outcome assessment, adjustment for confounding variables, and potential differences in demographic and lifestyle characteristics.

We also found an inverse association between prenatal AA and allergy in children, and other observations were in line with our finding [36]. As an important component of cell membrane, AA can affect the function of ion channels and the activity of various enzymes, thereby exerting a vital impact on the health of embryos and infants [37,38]. In general, the metabolites of AA, including eicosanoid precursors such as prostaglandins (PG), thromboxane (TX) and leukotrienes (LT), are suggested to enhance allergic inflammation by increasing vascular permeability and eosinophil recruitment [39]. However, the AA-derived metabolites (e.g., lipoxin A4) contribute to the production of resolvins, which help resolve inflammation and promote wound healing [40]. Although excess AA might increase the risk of allergic diseases [41,42], we hypothesize that appropriate levels of AA may reduce the risk. Compared with other ethnic populations, Chinese have different PUFA composition, with lower levels of AA [43]. Furthermore, it is also critical to maintain a balance between *n*-6 PUFA and *n*-3 PUFA, because of their different regulatory roles in inflammatory response. In our study, the level of *n*-6/*n*-3 in the any allergic disease group was 3.68, which was between the optimal ratio of 2.3/1 recommended by American experts and 4/1 recommended by Japanese experts [44]. Given the reasons above, we believe that maternal AA at moderate concentration in this study can help reduce the risk of allergy in offspring.

In the subgroup analysis, we found that children born to women with an allergy history were more likely to benefit from higher prenatal total PUFA, *n*-6 PUFA and LA levels than their counterparts. However, the underlying mechanism of this finding is still unclear. There are studies demonstrating that children with a maternal allergy history have a genetic predisposition to allergy [45], and the PUFA metabolic pathway may be dysregulated in the allergic population [46], where moderate concentrations of PUFA may play a role. Future studies are needed to further verify the modification effect of maternal allergy history on antenatal PUFA and offspring allergy. We also found a positive association between DPA and risk of offspring allergy in the subgroup with allergy history. DPA serves as a repository for DHA and EPA and contributes to the maintenance of EPA and DHA levels, which is important for maintaining the *n*-3 LCPUFA level [47]. It also has been found that a higher level of maternal DPA was associated with better early development, such as fewer allergic reactions and better neural development [47]. Although a previous review [48] suggested that DPA could inhibit the production of inflammatory eicosanoids by competing with AA for cyclooxygenase, the mechanism of DPA in the immune process remains to be further studied.

Our prospective birth cohort study adds important insight into the relationship be-tween maternal prenatal PUFA and allergy in offspring in the Chinese population. Potential limitations should also be considered. Firstly, similarly to other observational studies, there is the possibility of unmeasured confounding. However, we have taken into account various maternal and child lifestyle characteristics in our analyses. Furthermore, we did not find robust evidence of sociodemographic confounding in our analyses. Secondly, as with most cohort studies, a limitation of the present study is dropout. Although attrition was almost inevitable, we tried to keep the dropout rate as low as possible by training investigators and streamlining questions. Thirdly, the lack of obvious allergic symptoms may introduce the possibility of underestimation of disease incidence. In order to reduce such underestimation, our questionnaire was adapted from the validated ISAAC study [24]. Additionally, detailed information on allergy was collected by trained investigators, which helped determine allergy outcomes more reliably. Fourthly, due to the limited length of follow-up in our study, we were unable to determine the potential long-term effect of maternal erythrocyte PUFA during pregnancy on allergic diseases in offspring. However, we expect to further investigate the association between early life PUFA and later-onset allergy in the future since our cohort is still ongoing. Finally, we did not measure erythrocyte fatty acids at multiple times during pregnancy, but only in the second trimester. Nevertheless, the dietary pattern in the second and third trimesters is relatively stable, and the red blood cell membrane fatty acids in the second trimester may be representative.

Based on our findings, we recommend that pregnant women consume foods rich in *n*-3 PUFA, such as fatty fish, algae, flax seeds, chia seeds and walnuts [49], in accordance with local dietary guidelines for pregnant women. However, dietary intake may not necessarily meet maternal and fetal needs. Therefore, *n*-3 PUFA supplementation, such as rich oil derived from microalgae schizochytrium species and pregnancy formula, may be considered. From a practical perspective, it needs to be further investigated whether fatty acids from different dietary sources have differences in metabolism and function within the body. Additionally, more evidence is required to determine which kinds of *n*-3 PUFA supplementation provide allergy protection in offspring. Finally, when and how to administer supplementation for maximum benefit also need to be investigated.

## 5. Conclusions

In conclusion, our study suggested that maternal erythrocyte PUFA, especially AA and *n*-3 PUFA, were adversely associated with allergic diseases in the first 2 years of life. Moreover, there was a significant interaction between maternal allergy history and antenatal *n*-6 PUFA in offspring allergy.

## Figures and Tables

**Table 1:** Characteristics of the study population (n, %).

Characteristics	Total	No Allergic Disease	Any Allergic Disease	*p* Value
	*N =* 573	*N =* 336	*N =* 237	
Maternal general characteristics				
Maternal age (>35 years)	109 (19.02)	63 (18.75)	46 (19.41)	0.928
BMI (kg/m^2^)				0.595
Normal or thin	504 (87.96)	293 (87.20)	211 (89.03)	
Overweight or obese	69 (12.04)	43 (12.80)	26 (10.97)	
Educational level				0.151
Senior high school or below	198 (34.55)	125 (37.20)	73 (30.80)	
Junior college	176 (30.72)	103 (30.65)	73 (30.80)	
College or above	182 (31.76)	97 (28.87)	85 (35.86)	
Occupation				0.660
Administrators and clerks	123 (21.47)	75 (22.32)	48 (20.25)	
Commerce and services	160 (27.92)	97 (28.87)	63 (26.58)	
Housewives	139 (24.26)	83 (24.70)	56 (23.63)	
Others	129 (22.51)	70 (20.83)	59 (24.89)	
Monthly household income (RMB)				0.165
<4000 (about USD 626)	109 (19.02)	70 (20.83)	39 (16.46)	
4000–10,000(about USD 626–1567)	263 (45.90)	157 (46.73)	106 (44.73)	
>10,000 (about USD 1567)	180 (31.41)	96 (28.57)	84 (35.44)	
Frequency of passive smoking				**0.037**
0 day	**418 (72.95)**	**254 (75.60)**	**164 (69.20)**	
1–2 days a week	**67 (11.69)**	**38 (11.31)**	**29 (12.24)**	
3–5 days a week	**24 (4.19)**	**8 (2.38)**	**16 (6.75)**	
Almost every day	**29 (5.06)**	**16 (4.76)**	**13 (5.49)**	
Unclear	**16 (2.79)**	**6 (1.79)**	**10 (4.22)**	
Regular drinking				0.052
No	546 (95.29)	325 (96.73)	221 (93.25)	
Yes	22 (3.84)	8 (2.38)	14 (5.91)	
Maternal allergy history				**0.004**
No	**506 (88.31)**	**308 (91.67)**	**198 (83.54)**	
Yes	**67 (11.69)**	**28 (8.33)**	**39 (16.46)**	
Offspring’s general characteristics				
Gender of infant				0.520
Male	267 (46.60)	150 (44.64)	117 (42.86)	
Female	263 (45.90)	156 (46.43)	107 (45.15)	
Breastfeeding duration (months)				**0.034**
<4	**239 (41.71)**	**130 (38.69)**	**109 (45.99)**	
4–6	**99 (17.28)**	**54 (16.07)**	**45 (18.99)**	
≥6	**186 (32.46)**	**123 (36.61)**	**63 (26.58)**	
Complementary feeding (months)				0.971
<6	462 (80.63)	269 (80.06)	193 (81.43)	
≥6	76 (13.26)	45 (13.39)	31 (13.08)	

NOTE: Statistically significant results are in bold (*p* < 0.05).

**Table 2:** Maternal erythrocyte fatty acids during pregnancy between the allergic disease group and the control group ^a^.

Erythrocyte Fatty Acids (%)	No Allergic Disease	Any Allergic Disease	*p* Value
	*N* = 336	*N* = 237	
Total PUFA	**45.92 (43.06, 48.54)**	**45.42 (42.49, 47.77)**	**0.044**
*n*-6-PUFA	36.27 (33.54, 38.31)	36.24 (33.20, 38.07)	0.534
AA	**17.58 (15.12, 19.11)**	**17.07 (14.33, 18.72)**	**0.042**
LA	15.29 (13.91, 16.68)	15.50 (13.79, 17.06)	0.268
GLA	0.22 (0.14, 0.31)	0.24 (0.15, 0.32)	0.131
DGLA	2.45 (2.09, 2.77)	2.41 (2.05, 2.81)	0.673
*n*-3-PUFA	**10.01 (7.86, 12.08)**	**9.43 (7.75, 10.91)**	**0.011**
ALA	0.24 (0.18, 0.33)	0.24 (0.18, 0.35)	0.739
EPA	0.96 (0.66, 1.36)	0.87 (0.63, 1.37)	0.459
DHA	7.24 (5.42, 9.10)	6.91 (5.07, 8.75)	0.319
DPA	1.25 (1.00, 1.53)	1.23 (1.02, 1.51)	0.519
Omega-3 Index	8.18 (6.51, 10.19)	8.11 (6.02, 9.68)	0.276
*n*-6/*n*-3	3.59 (3.08, 4.21)	3.68 (3.15, 4.22)	0.259
AA/EPA	17.66 (11.67, 25.80)	18.65 (11.04, 26.17)	0.959

NOTE: Statistically significant results are in bold (*p* < 0.05). ^a^ Values were presented as median (Q_1_, Q_3_). Abbreviation: PUFA, polyunsaturated fatty acids; AA, arachidonic acid; LA, linoleic acid; GLA, *γ*-linolenic acid; DGLA, *Dihomo*-*γ*-linolenic; ALA, *α*-linolenic acid; EPA, eicosapentaenoic acid; DHA, docosahexaenoic acid; DPA, docosapentaenoic acid; Omega-3 index (O3I) is the sum of erythrocyte EPA and DHA. *n*-6/*n*-3 is the ratio of *n*-3 to *n*-6 PUFA; AA/EPA is the ratio of AA to EPA.

**Table 3:** The associations between maternal erythrocyte fatty acids and offspring allergic disease ^a^ within 2 years of age.

Erythrocyte Fatty Acids (*N* = 573)	MODEL1		MODEL2	
	*HR* (95% CI)	*p* Value	*HR* (95% CI)	*p* Value
Total PUFA	**0.87 (0.75, 0.99)**	**0.040**	**0.80 (0.68, 0.94)**	**0.008**
*n*-6-PUFA	0.95 (0.83, 1.09)	0.487	0.88 (0.75, 1.04)	0.126
AA	0.87 (0.73, 1.03)	0.106	**0.79 (0.65, 0.97)**	**0.025**
LA	1.06 (0.91, 1.24)	0.425	1.02 (0.84, 1.23)	0.840
GLA	1.02 (0.96, 1.09)	0.540	1.02 (0.95, 1.10)	0.563
DGLA	0.98 (0.83, 1.15)	0.796	0.88 (0.73, 1.07)	0.201
*n*-3-PUFA	**0.78 (0.65, 0.94)**	**0.010**	**0.77 (0.62, 0.97)**	**0.024**
ALA	0.99 (0.96, 1.03)	0.746	0.99 (0.95, 1.03)	0.751
EPA	0.94 (0.79, 1.13)	0.538	0.95 (0.76, 1.18)	0.629
DHA	0.92 (0.77, 1.10)	0.376	0.81 (0.65, 1.01)	0.059
DPA	0.96 (0.86, 1.06)	0.376	1.00 (0.89, 1.12)	0.995
Omega-3 Index	0.92 (0.77, 1.09)	0.343	0.82 (0.67, 1.01)	0.064
*n*-6/*n*-3	1.06 (0.91, 1.23)	0.473	1.09 (0.91, 1.31)	0.323
AA/EPA	1.02 (0.85, 1.22)	0.863	0.99 (0.79, 1.24)	0.933

NOTE: Statistically significant results are in bold (*p* < 0.05). The results in the table are the *HR* value and 95% *CI* corresponding to original value/IQR in erythrocyte fatty acids. ^a^ 41.36% share (237/573) of children had any allergic disease, including eczema, atopic dermatitis, urticaria, allergic rhinitis, allergic conjunctivitis, pollen allergy, food allergy, and asthma. MODEL1: Not adjusted. MODEL2: Adjusted for maternal age, maternal BMI, educational level, occupation, monthly household income, gender of infant, breastfeeding duration, mother’s allergy history, complementary feeding time, maternal passive smoking and maternal alcohol consumption.

**Table 4:** The associations between maternal erythrocyte fatty acids and offspring allergic disease ^a^ within 2 years of age, stratified by maternal allergy history and maternal age.

Erythrocyte Fatty Acids (*N* = 573)	Maternal Allergy History [*HR (95% CI)*]			Maternal Age [*HR (95% CI)*]		
	NO	YES	*p* ^b^	≤35 Years	>35 Years	*p* ^b^
Total PUFA	**0.80 (0.67, 0.96)**	**0.39 (0.21, 0.72)**	**0.028**	**0.75 (0.63, 0.89)**	1.09 (0.64, 1.84)	0.190
*n*-6-PUFA	0.91 (0.76, 1.08)	**0.41 (0.23, 0.75)**	**0.013**	**0.83 (0.70, 0.98)**	1.07 (0.70, 1.65)	0.273
AA	0.80 (0.64, 0,99)	0.55 (0.25, 1.18)	0.357	**0.79 (0.63, 0.98)**	0.78 (0.46, 1.34)	0.975
LA	1.09 (0.88, 1.34)	**0.45 (0.25, 0.81)**	**0.006**	0.90 (0.73, 1.11)	1.30 (0.88, 1.91)	0.107
GLA	1.05 (0.98, 1.13)	0.81 (0.59, 1.13)	0.135	1.03 (0.95, 1.11)	1.11 (0.73, 1.68)	0.726
DGLA	0.88 (0.72, 1.09)	0.50 (0.24, 1.04)	0.139	**0.79 (0.64, 0.98)**	1.03 (0.62, 1.71)	0.349
*n*-3-PUFA	**0.74 (0.58, 0.94)**	0.85 (0.37, 1.93)	0.760	**0.72 (0.56, 0.93)**	0.98 (0.57, 1.70)	0.313
ALA	0.99 (0.95, 1.03)	0.99 (0.83, 1.17)	0.954	0.99 (0.95, 1.04)	1.11 (0.92, 1.34)	0.248
EPA	0.95 (0.75, 1.21)	0.87 (0.37, 2.00)	0.827	0.94 (0.74, 1.19)	1.10 (0.68, 1.78)	0.554
DHA	0.81 (0.65, 1.03)	0.77 (0.37, 1.59)	0.879	0.79 (0.62, 1.01)	0.83 (0.49, 1.42)	0.870
DPA	0.97 (0.85, 1.10)	**1.50 (1.08, 2.08)**	0.015	1.01 (0.89, 1.15)	0.94 (0.68, 1.31)	0.682
Omega-3 Index	0.83 (0.66, 1.03)	0.77 (0.38, 1.55)	0.850	0.80 (0.64, 1.01)	0.87 (0.53, 1.43)	0.782
*n*-6/*n*-3	1.16 (0.94, 1.43)	0.71 (0.43, 1.18)	0.079	1.08 (0.88, 1.32)	1.15 (0.76, 1.74)	0.779
AA/EPA	0.99 (0.77, 1.26)	0.98 (0.45, 2.14)	0.996	0.98 (0.76, 1.25)	0.82 (0.46, 1.46)	0.586

NOTE: Statistically significant results are in bold (*p* < 0.05). ^a^ 41.36% share (237/573) of the children had any allergic disease, including eczema, atopic dermatitis, urticaria, allergic rhinitis, allergic conjunctivitis, pollen allergy, food allergy, and asthma. ^b^ *p* for difference of effect between subgroups. Cox model was adjusted for maternal age, maternal BMI, educational level, occupation, monthly household income, gender of infant, breastfeeding duration, mother’s allergy history, complementary feeding time, maternal passive smoking and maternal alcohol consumption (except for stratified factor, respectively).

## Data Availability

Due to ethical requirements, the datasets presented in this article are not publicly available, but available on request from the corresponding author.

## Supplementary Materials

The following supporting information can be downloaded at: https://www.mdpi.com/article/10.3390/nu14112312/s1, Table S1: The associations between maternal erythrocyte fatty acids and offspring specific allergic diseases within 2 years of age.
