# Supplementary material for: Association of Maternal Erythrocyte PUFA during Pregnancy with Offspring Allergy in the Chinese Population
Source: Nutrients. 2022 May 31;14(11):2312. doi: 10.3390/nu14112312 (PMC9182582; doi:10.3390/nu14112312)
Supplement: Supplementary file 1 [file nutrients-14-02312-s001.zip › nutrients-1721275-supplementary.pdf]

Table S1. The associations between maternal erythrocyte fatty acids and offspring specific allergic diseases within 2 years old.

Erythrocyte fatty acids	Specific allergic diseases [*HR (95% CI)*]			
	**Eczema (*n*=181)**	**Urticaria (*n*=33)**	**Food Allergy (*n*=57)**	**Allergic Rhinitis (*n*=22)**
PUFA	**0.81 (0.67, 0.98)**	0.92 (0.60, 1.40)	0.82 (0.60, 1.12)	0.91 (0.51, 1.61)
n-6-PUFA	0.90 (0.75, 1.08)	0.88 (0.60, 1.31)	0.87 (0.64, 1.17)	1.03 (0.58, 1.83)
AA	0.83 (0.66, 1.04)	0.84 (0.50, 1.38)	**0.65 (0.44, 0.96)**	0.94 (0.46, 1.95)
LA	1.02 (0.82, 1.26)	0.96 (0.59, 1.55)	1.22 (0.84, 1.78)	1.25 (0.65, 2.41)
GLA	1.03 (0.95, 1.12)	0.99 (0.78, 1.25)	1.08 (0.97, 1.20)	0.99 (0.78, 1.25)
DGLA	0.85 (0.68, 1.05)	1.05 (0.65, 1.69)	0.85 (0.58, 1.22)	0.97 (0.52, 1.80)
n-3-PUFA	**0.77 (0.60, 0.99)**	1.10 (0.62, 1.94)	0.82 (0.51, 1.33)	0.70 (0.30, 1.62)
ALA	1.01 (0.97, 1.05)	1.01 (0.92, 1.12)	1.00 (0.93, 1.08)	0.96 (0.80, 1.15)
EPA	0.82 (0.64, 1.06)	1.17 (0.69, 1.99)	1.17 (0.77, 1.76)	1.77 (0.90, 3.45)
DHA	0.80 (0.63, 1.03)	0.88 (0.51, 1.52)	0.80 (0.52, 1.24)	1.20 (0.56, 2.57)
DPA	1.01 (0.88, 1.15)	0.92 (0.68, 1.24)	0.87 (0.70, 1.08)	0.95 (0.65, 1.38)
Omega-3 Index	0.80 (0.63, 1.01)	0.92 (0.55, 1.54)	0.84 (0.55, 1.28)	1.32 (0.64, 2.70)
n-6/n-3	1.11 (0.91, 1.36)	0.93 (0.56, 1.54)	1.18 (0.83, 1.69)	0.91 (0.47, 1.77)
AA/EPA	1.06 (0.83, 1.36)	0.94 (0.52, 1.67)	0.85 (0.54, 1.34)	0.72 (0.33, 1.57)

NOTE： Statistically significant results are in bold ($P < 0.05$).

The results in the table are the HR value and 95%CI corresponding to original value/IQR in erythrocyte fatty acid.

MODEL： Adjusted for maternal age， maternal BMI, educational level，occupation, monthly household income, gender of infant, breastfeeding duration, mother's. allergy history, complementary feeding time, maternal passive smoking and maternal alcohol consumption.
